# Supplementary material for: CD177 modulates the function and homeostasis of tumor-infiltrating regulatory T cells
Source: Nat Commun. 2021 Oct 1;12:5764. doi: 10.1038/s41467-021-26091-4 (PMC8486774; doi:10.1038/s41467-021-26091-4)
Supplement: Supplementary file 1 — Supplementary Information [file 41467_2021_26091_MOESM1_ESM.pdf]

Supplementary information:

Title: CD177 modulates the functions and homeostasis of tumor-infiltrating regulatory T cells

First Authors: Myung-Chul Kim, DVM, Nicholas Borcherding, MD/PHD/MS, Kawther K. Ahmed, PHD, Andrew P. Voigt, BA

Corresponding Authors: Xuefeng Wu, PHD, Yousef Zakharia, MD, Weizhou Zhang, PHD

Supplementary Figures

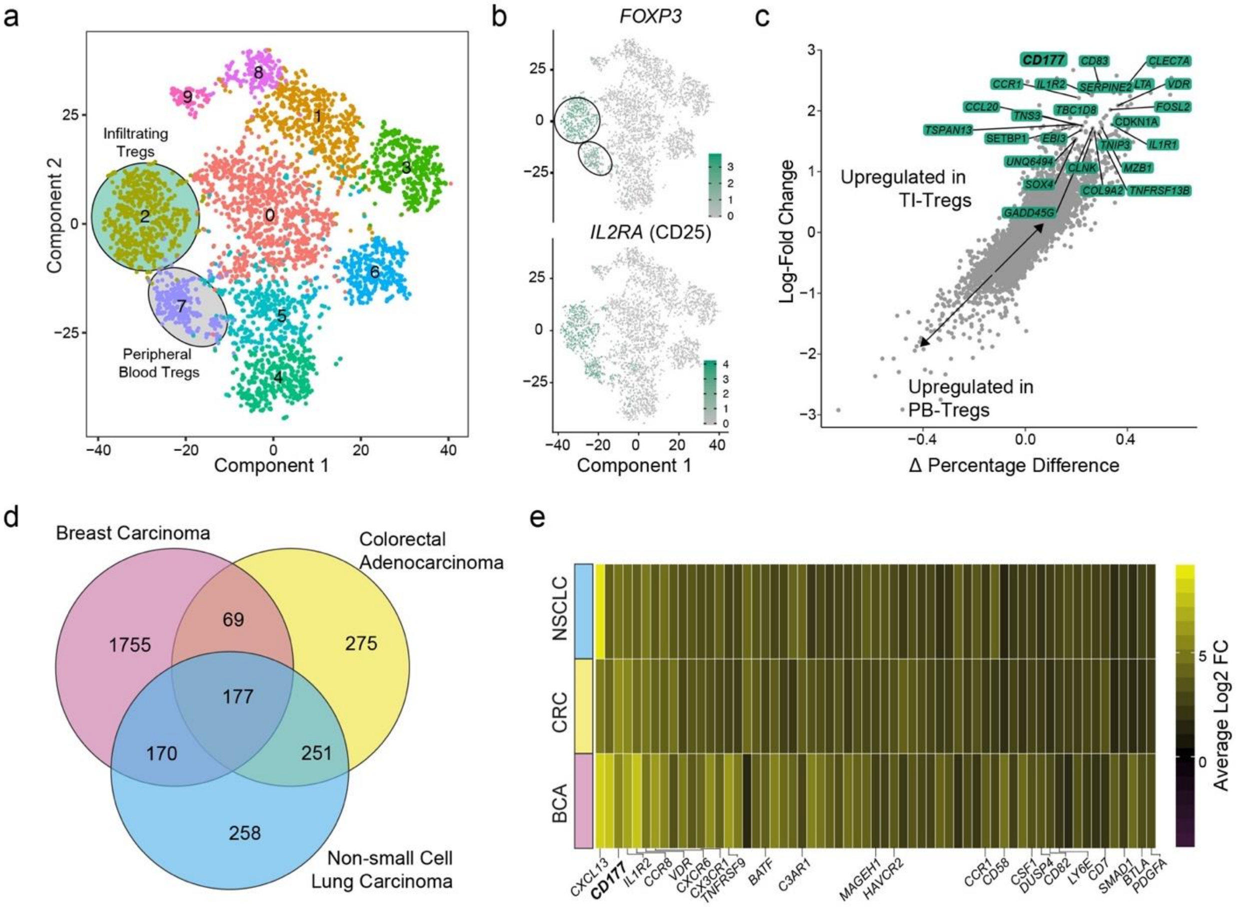

Supplementary Figure 1. Differential gene expression of TI-Treg cells in single-cell RNA sequencing and pooled RNA sequencing. **a.** tSNE projection of T cells from five HCC patients with normal PB cells and TI cells. TI Treg population (green, n=634) and PB-Treg population (grey, n=264) were isolated for further analysis. **b.** tSNE projection with highlighted expression of Treg markers, *FOXP3* and *IL2RA* (CD25). **c.** Differential gene expression analysis using the log-fold change expression versus the difference in the percent of cells expressing the gene comparing TI- versus PB-Treg cells (Δ Percentage Difference). **d.** Venn diagram showing the overlap in differentially-expressed genes in TI Treg cells compared to PB-Treg cells in breast carcinoma (GSE89225), colorectal adenocarcinoma (PRJEB11844), and non-small cell lung carcinoma (PRJEB11844). **e.** Heatmap showing differential genes shared between the three datasets with immune-related genes labeled. Genes are displayed in log2-fold change comparing TI Treg cells *versus* PB Treg cells with FDR q-values <0.05.

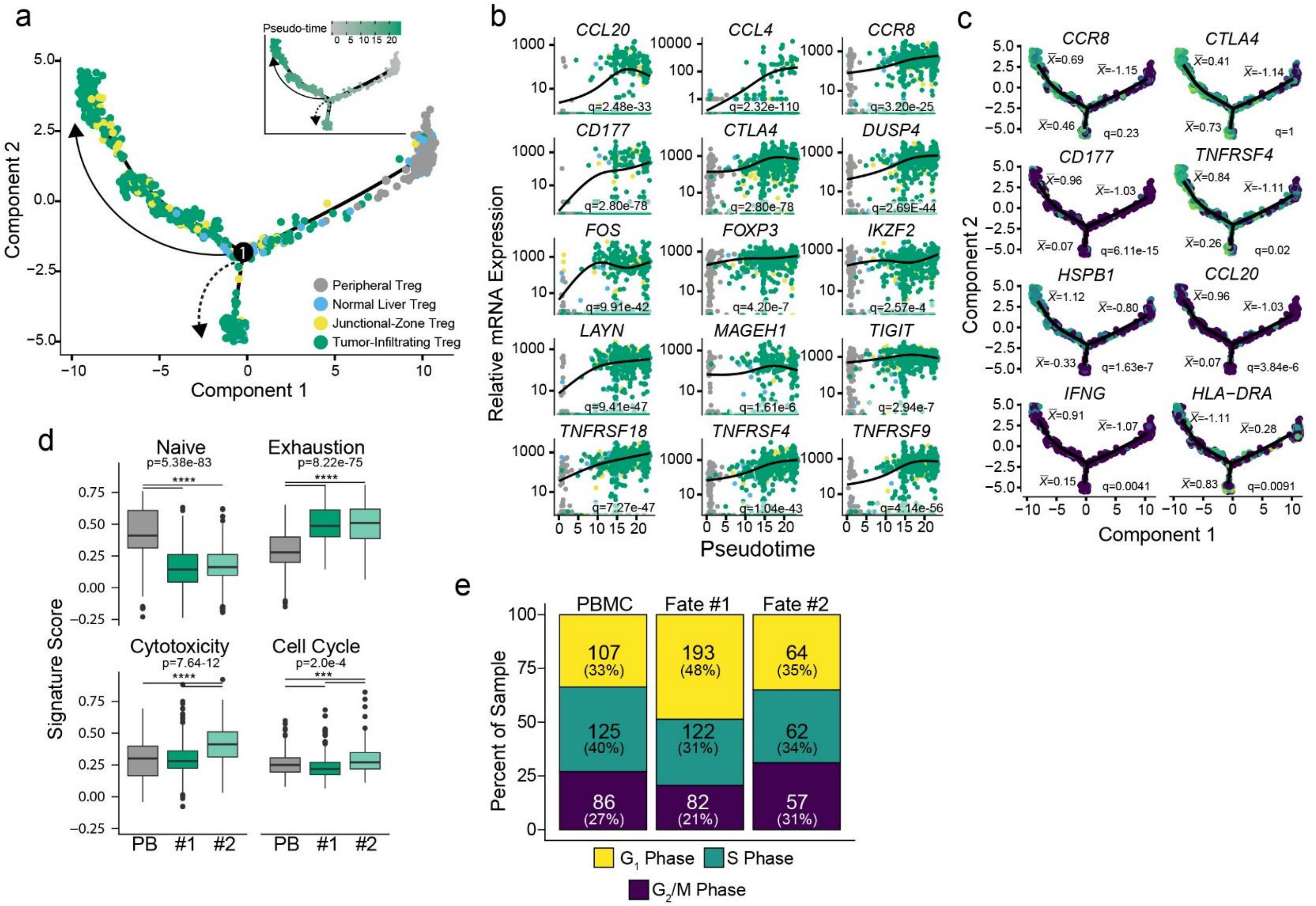

Supplementary Figure 2. Transcriptional heterogeneity in single-cell RNAseq of HCC Treg cells. a. Trajectory manifold of Treg cells from the HCC using the Monocle 2 algorithm. **b.** Pseudotime projections of transcriptional changes in immune genes based on the manifold. **c.** Cell trajectory projections of transcriptional changes in immune genes based on the manifold. $\bar{x}$ denotes the scaled mean of each pole of the manifold. **d.** Gene signature analysis of the poles of the trajectory manifold. Boxplot plot drawn for values between 25th and 75th percentile, with median value lines. Outlier values were graphed as individual points for values 1.5 times the interquartile range. *P*-values are based on one-way ANOVA with individual comparisons corrected for multiple hypothesis testing using the Tukey HSD method. **e.** Results of the cell cycle regression analysis of single cells for each cell fate using the Seurat R package.

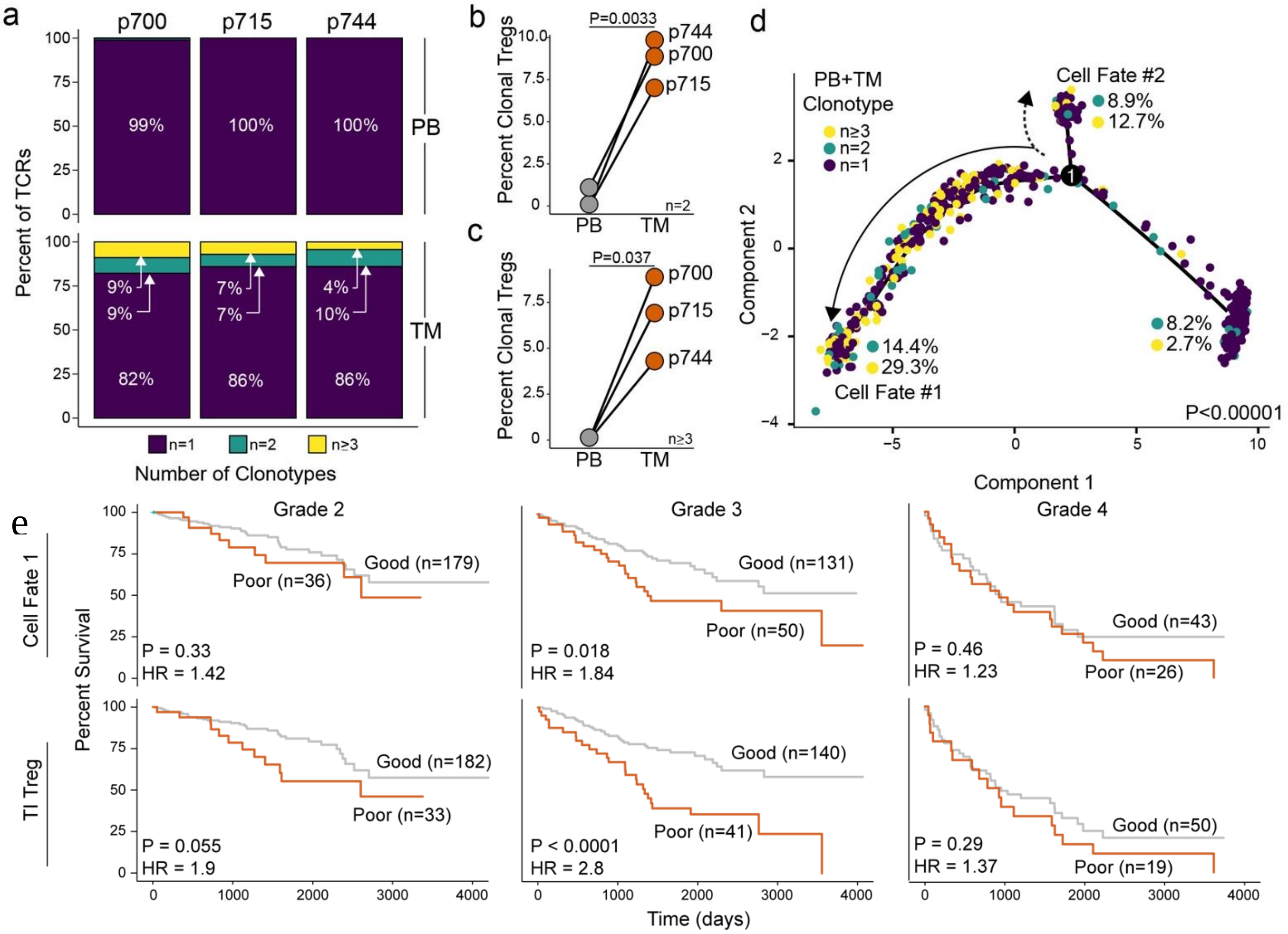

Supplementary Figure 3. Clonotype analysis of TI- or PB-Treg cells. a. Percentages of assigned clonotypes by patients in PB- (upper panel) and TI-Treg cells (TM, lower panel). **b.** Relative increase in clonotypes with two copies in the same patient comparing PB-Treg cells (grey) to TI-Treg cells (orange). Significance testing utilized T test with Welch's correction. **c.** Relative increase in clonotypes with three or more copies in the same patient comparing PB-Treg cells (grey) to TI-Treg cells (orange). **b-c**: two-sided unpaired T-test **d.** Cell trajectory projections of shared clonotype percentages by cell fate, combining the PB- and TI-Treg cells clonotypes of a single patient. Significance based on χ^2 testing. **e.** Kaplan-Meier curves for overall survival in the TCGA KIRC/ccRCC using the CF1-Treg gene signature (upper panel) and TI-Treg common gene signature (lower panel) by the histological grades. Logrank test for trend was used for **e**.

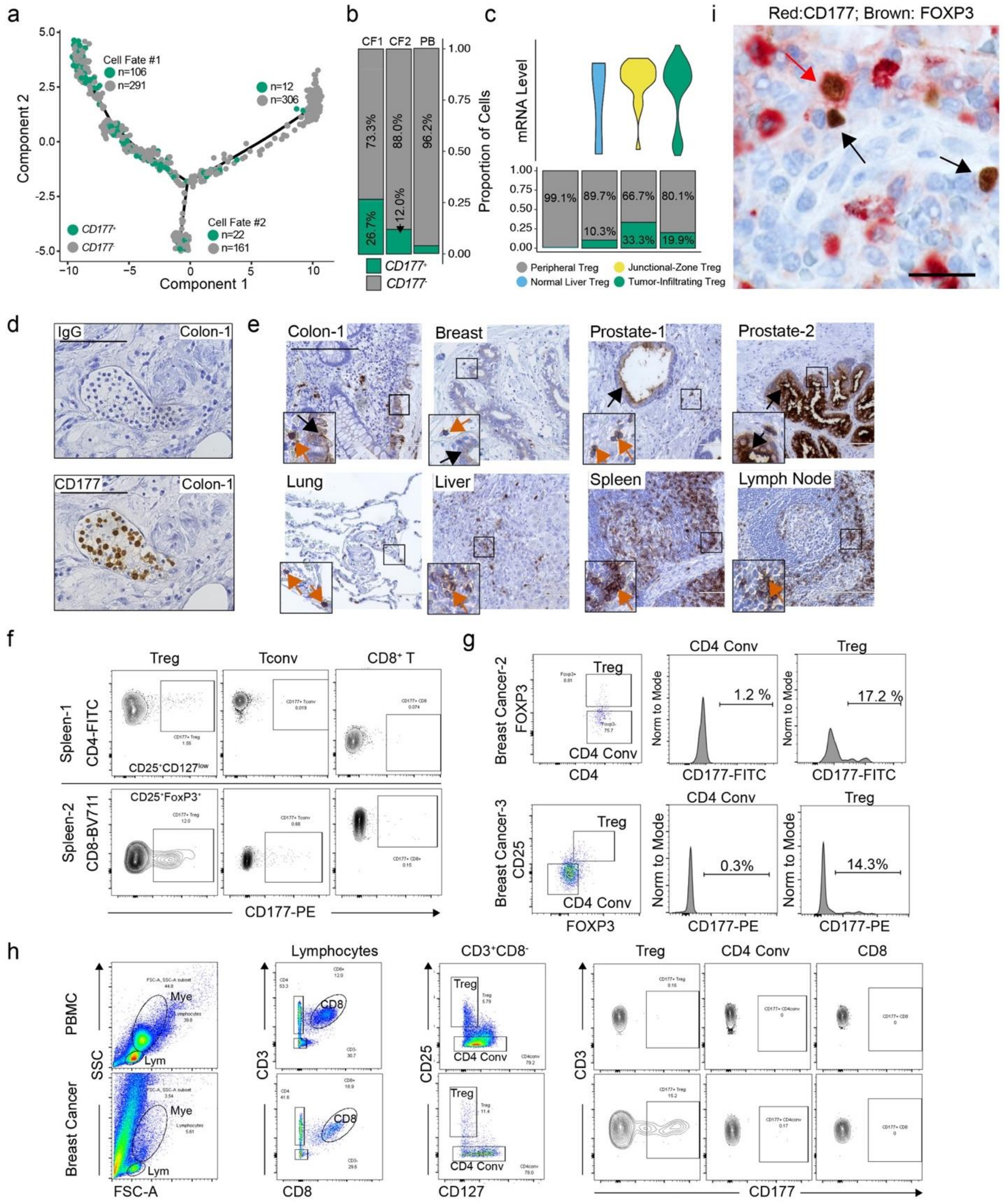

Supplementary Figure 4. CD177$^+$ TI lymphocytes are comprised of Treg cells. a. Trajectory manifold of Treg cells from HCC or PB with the number of CD177$^+$ and CD177$^-$ Treg cells for each respective cell fate. Significance based on χ^2 testing comparing the three poles of the manifold. **b.** Proportional distribution of *CD177*$^+$ Treg cells by cell fate across the manifold from HCC. **c.** Violin plot showing mRNA expression of *CD177* (upper panel) or Proportional distribution of *CD177*$^+$ Treg cells from HCC, junctional zone, normal liver, or PB (lower panel). **d.** IHC staining protocol is established and the specificity of anti-CD177 antibody (clone: 4C4) is confirmed using isotype IgG on sequential sections of normal human colon tissue, scale bar = 100μm. Note colon-associated granulocytes were evident for CD177 positivity (lower panel) (n = 2 biological repeats). **e.** CD177 IHC staining on different normal human tissues showing CD177 expression on normal epithelium for select tissues as well as infiltrating leukocytes. Black arrows: epithelial cells; orange arrows: lymphocytes scale; bar = 200μm. (n = 2 biological repeats) **f**. CD177 protein expression within human splenocytes. Two human

splenocytes from cancer patients (spleen-1 from melanoma and spleen-2 from pancreatic cancer) were analyzed by flow cytometry to quantify CD177 expression within different T cell subsets. **g.** Two additional breast cancer specimens were further analyzed for CD177 expression on Treg cells (CD4+FoxP3^{+} or CD4+CD25^{+}FoxP3^{+}) or conventional CD4 T cells (CD4 conv, CD4^{+}CD25^{-}) from TI lymphocytes in breast cancer or PBMC lymphocytes. **h.** Schematic flow cytometry data gating on lymphocytes (lym) or myeloid cells (mye) were further analyzed for CD177 expression on Treg cells (CD4+CD25^{+}CD127^{-}), conventional CD4 T cells (CD4 conv, CD4^{+}CD25^{-}CD127$^{+/-}$) and CD8 T cells (CD8^{+}) isolated from TI immune cells in breast cancer or PBMC. Schematic gating is used for Figure 4d-e as well. **i.** Representative dual IHC staining for CD177 (red) and FoxP3 (brown) in breast carcinoma section. Dual-positive cells are indicated with red arrows and FoxP3-positive cells are indicated by black arrows (n = 3 biological repeats).

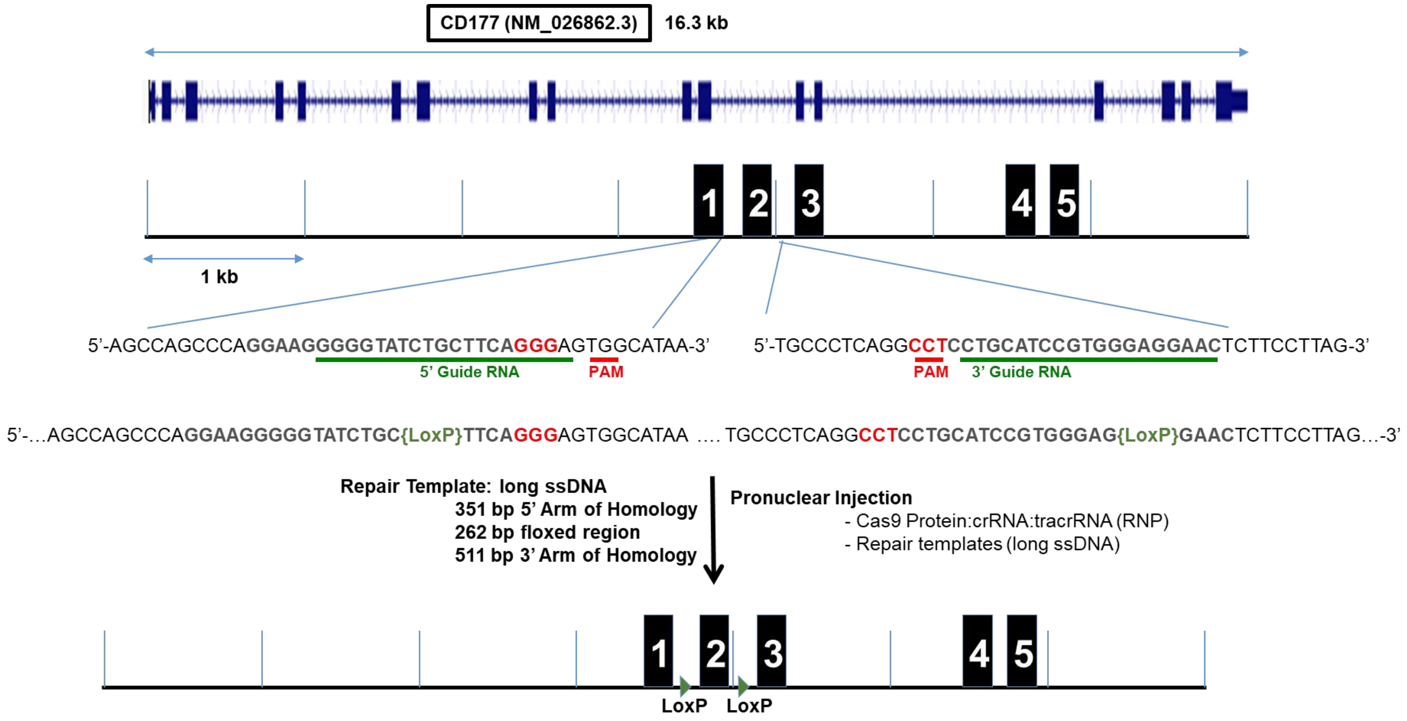

Supplementary Figure 5. Schematic targeting mouse *Cd177* gene locus using CRISPR/Cas9-based homologous recombination. Exon 2 was flanked by two LoxP sites and expected to be removed by cre DNA recombinase, which produces a stop codon after joining Exon 1 and Exon 3.

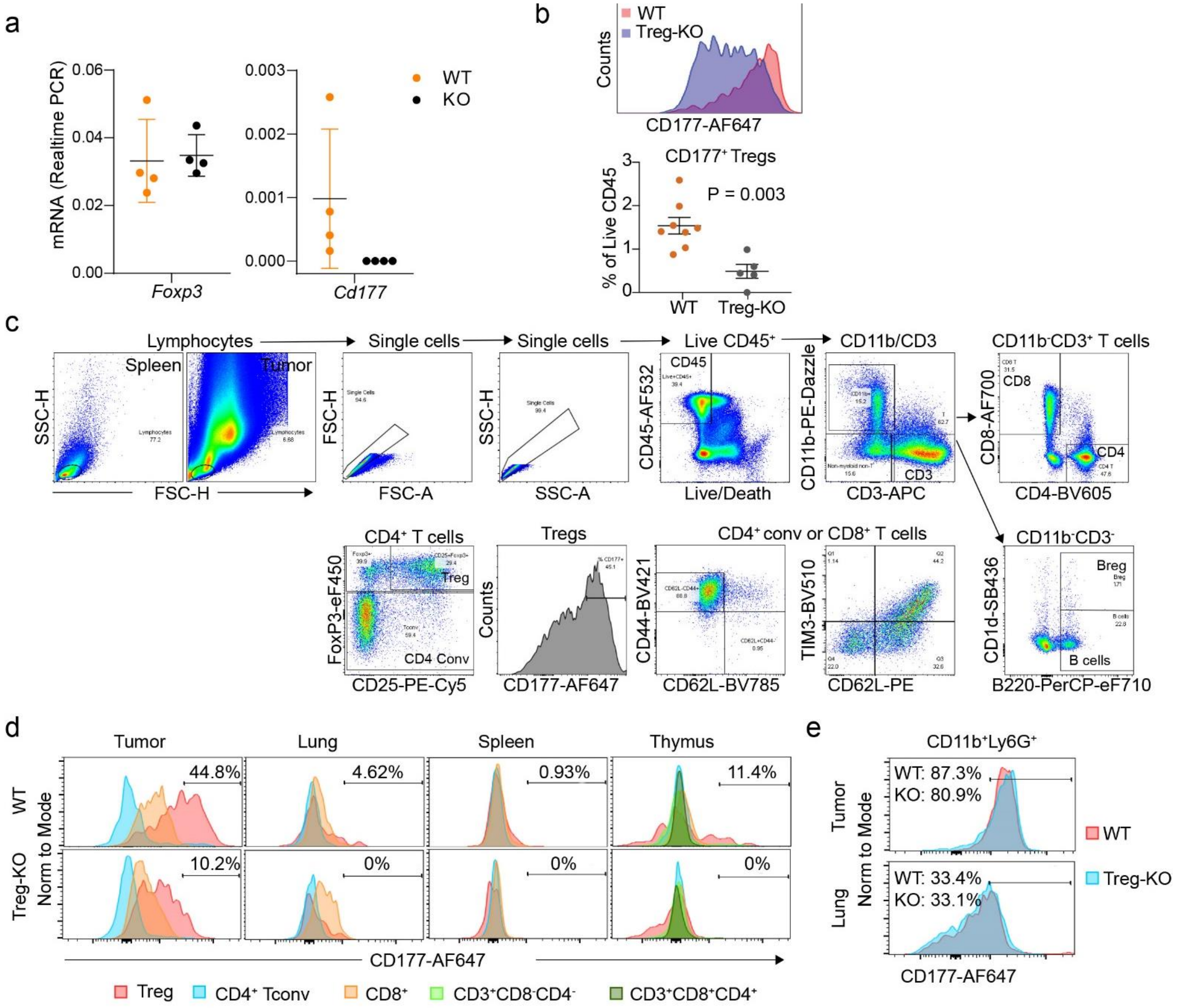

Supplementary Figure 6. Impact of Treg-specific *Cd177*-deficiency on immune cells within tumors or other tissues. **a.** Confirmation of *Cd177*-deficiency using real-time PCR analysis. Treg cells isolated from tumors from WT or KO mice in Figure 5b were subjected to mRNA isolation, reverse transcription, and real-time PCR analysis (n = 4) of *Foxp3* (left) or *Cd177* (right), using *Ppia* as the reference housekeeping gene. **b**. Depletion of TI-CD177$^+$ Treg cells within Treg-specific *Cd177*-KO (*Cd177$^{fl/fl}$/Foxp3-Cre)* mice (n = 5-8) from MC38-tumor-bearing mice in Figure 5c. **a-b**: All error bars represent mean ± s.t.d. **c.** Schematic gating strategy for analysis of mouse lymphocytes from tumor or other tissues used in **Figure 6a-c**; **Figure 7e; Supplementary Figures 7-8; 9b,d,h**). Lymphocyte gating from all tissues was guided by lymphocyte population from mouse splenocytes. **d-e.** CD177 expression from **(d)** T cells or **(e)** neutrophils within different tissues of tumor-bearing mice. MC38 tumor-bearing WT or Treg-specific *Cd177* KO (Treg-KO) mice were analyzed for CD177 protein expression from various T cell subtypes including Treg cells (CD4$^+$CD25$^+$FoxP3$^+$) or neutrophils (CD11b$^+$Ly6G$^+$) within different tissues.

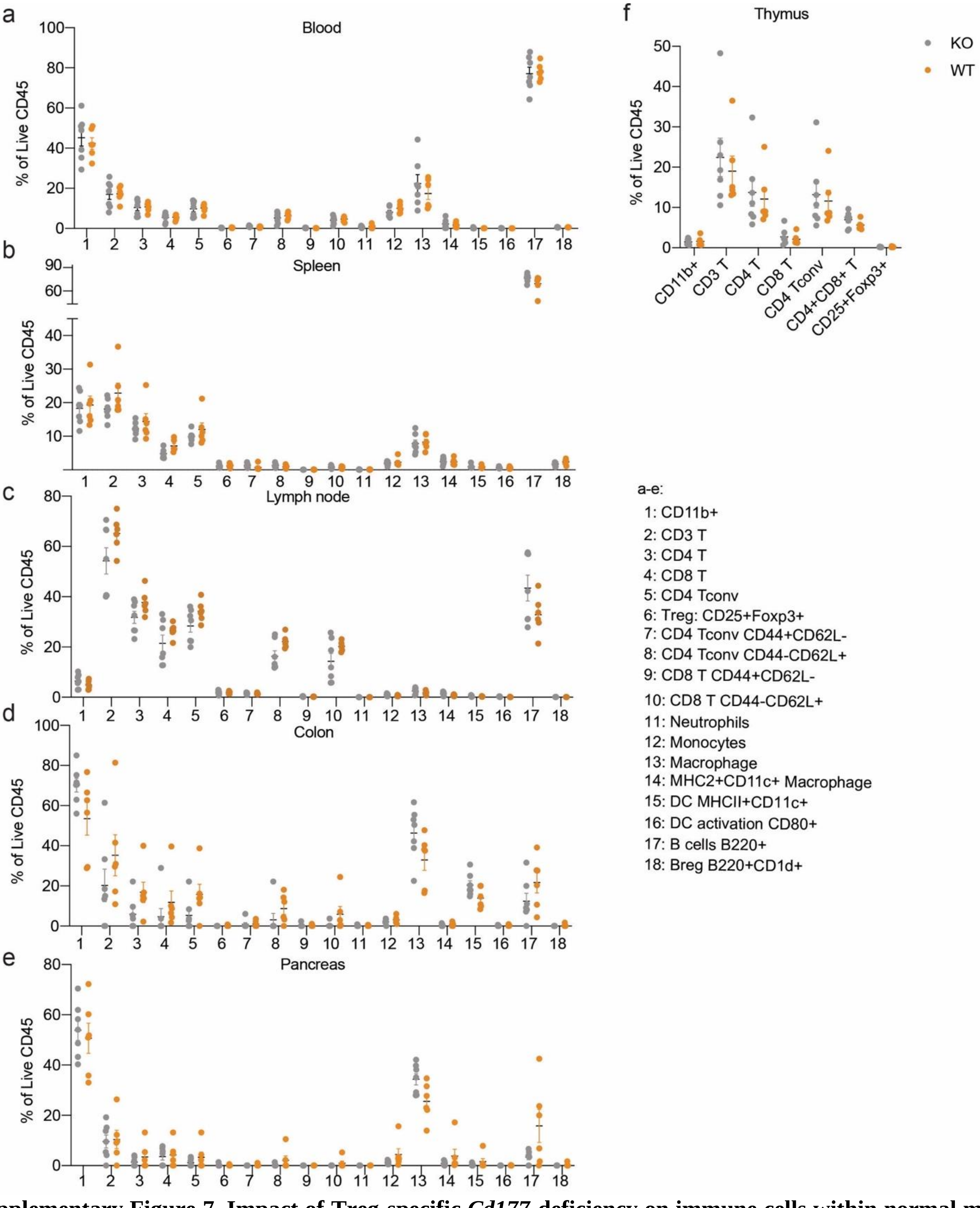

Supplementary Figure 7. Impact of Treg-specific *Cd177*-deficiency on immune cells within normal mice. 7-8-week-old mice – including 3-4 females and 3 males of WT or Treg-specific *Cd177* KO mice – were analyzed for different immune cell populations from **(a)** blood, **(b)** spleen, **(c)** lymph node, **(d)** colon, **(e)** pancreas, and **(f)** thymus. All data are presented mean ± s.t.d. Two-sided unpaired T-test was used for all group comparisons without adjustment for multiple comparisons.

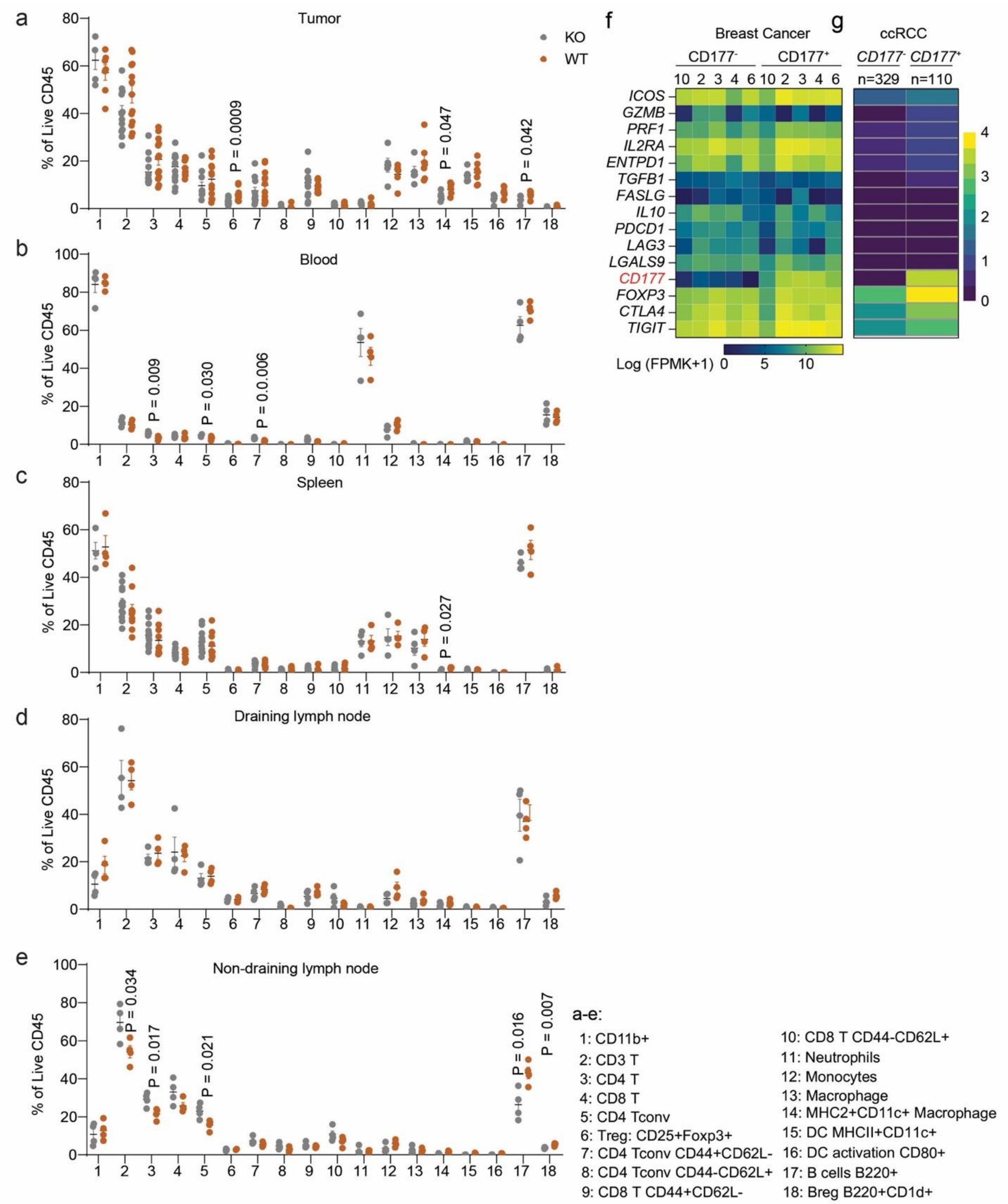

Supplementary Figure 8. Impact of Treg-specific *Cd177*-deficiency on immune cells within tumor-bearing mice. MC38 tumor-bearing WT or Treg-specific *Cd177* KO mice were analyzed for different immune cell populations from **(a)** tumor**, (b)** blood, **(c)** spleen, **(d)** draining or **(e)** non-draining lymph nodes. **f-g**. Heatmap showing Treg-relevant gene expression of CD177^{+} and CD177^{-} TI-Treg cells. CD177^{+} and CD177^{-} TI Treg cells from 5 human breast cancer specimens **(f,** data adapted from GSE89225 with patient numbers included) or from our own ccRCC SCRS dataset (**g**, based on *CD177* mRNA expression, GSE121638) were analyzed for Treg-relevant genes. All data are presented mean ± s.t.d. Two-sided unpaired T-test was used for all group comparisons without adjustment for multiple comparisons.

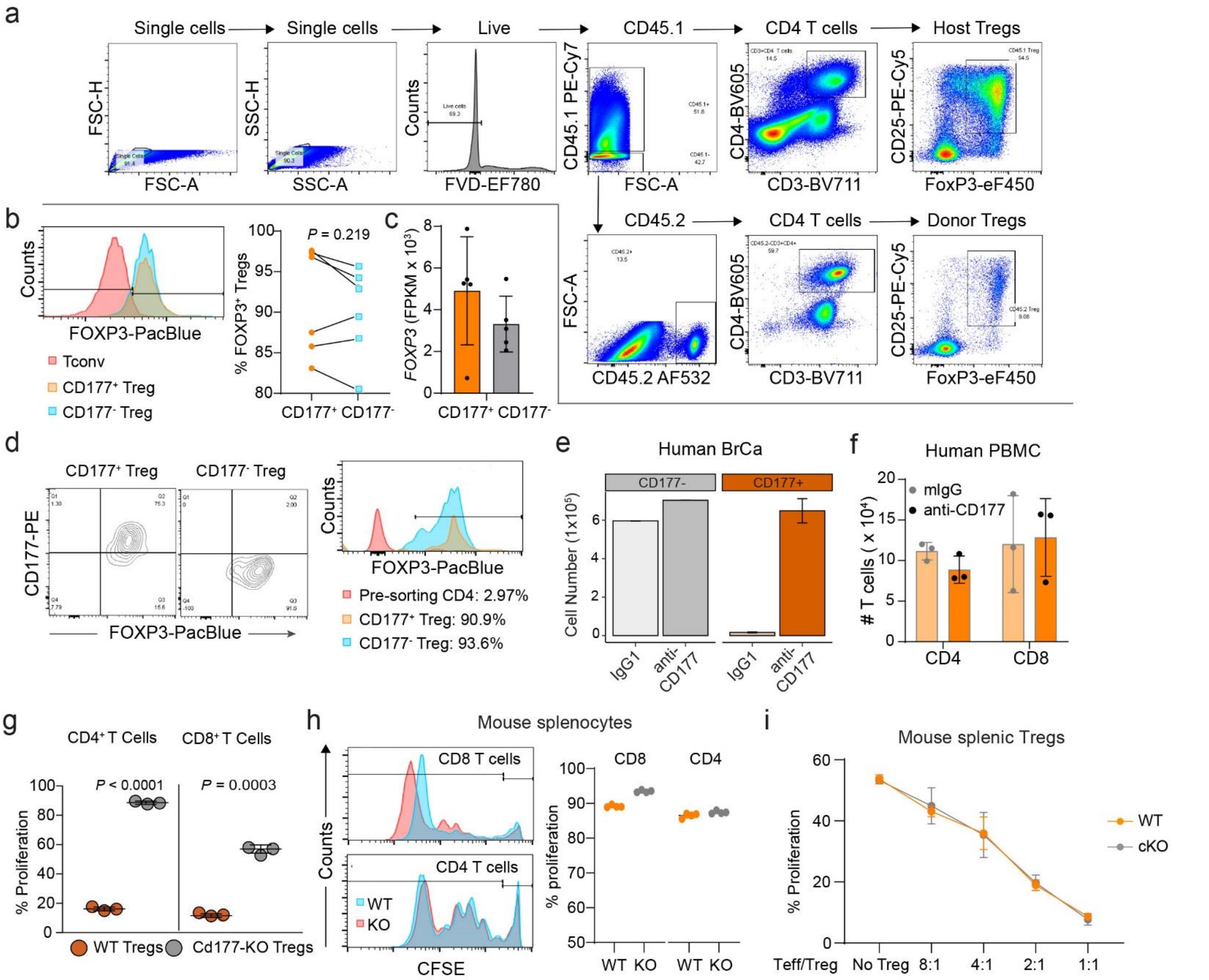

Supplementary Figure 9. Functional characterization of CD177⁺ TI-Treg cells. a. Schematic gating strategy for analysis of mouse lymphocytes from tumors for Fig. 6f-j. **b.** FOXP3 expression within CD177⁺ or CD177⁻ CD4⁺CD25⁺CD127^{low} Treg cells from human cancer specimens, including 1 breast cancer, 3 ccRCCs and 2 colon cancers. Two-sided paired T-test was used here. **c.** *FOXP3* mRNA expression of CD177⁺ and CD177⁻ TI Treg cells from 5 breast cancer patients, data adapted from GSE89225. **d**. Validation of post-sorting purity by flow cytometry – illustrated by FOXP3 positivity – of CD177⁺ and CD177⁻ TI Treg cells from breast cancer specimen. **e**. Impact of CD177 blockage on the immune suppressive function of CD177⁺ TI Treg cells. Total number of CD4 Tconv cells were enumerated (n=2, supplementary to Fig. 7b). **f.** Impact of anti-CD177 antibody on effector T cells. Effector CD4 and CD8 T cells from PBMC were purified and stimulated with anti-CD3 + APC with the presence of control IgG or anti-CD177 (MEM166) antibody. Total cells were enumerated after 4 days of incubation (n=3 biological replicates). **g.** Suppressive capacity of CD177⁺ or CD177⁻ TI Treg cells isolated from WT or germline CD177-KO mice bearing MC38 tumors as in Figure 5b. Effector CD4 T cells were stimulated by anti-CD3/CD28 co-stimulation and combined with TI Treg cells at 2:1 ratio (n = 3 biological replicates). **h.** Impact of CD177-deficiency on effector CD4 and CD8 T cells. Effector CD4 and CD8 T cells were purified from splenocytes of WT or germline CD177-KO mice, stimulated by anti-CD3 + APC. **Left:** Histograms showing the CFSE dilution as indicator of T cell proliferation and **right:** Percent proliferation as defined by the histogram (n=3 biological replicates). **i.** Impact of Treg-specific *Cd177*-KO (cKO) on the suppressive capacity of splenic Treg cells from non-tumor bearing mice (n = 3 biological replicates). All data are presented mean ± s.t.d. Two-sided unpaired T-test was used for all group comparisons without adjustment for multiple comparisons, unless otherwise indicated.

FC (mouse)	Clone	Company	Cat#	Color	Dilution
CD45.1	A20	BioLegend	110730	PE-CY7	1:100
CD45.2	30-F11	eBioscience	58-0451-82	AF532	1:100
CD45.2	104	BioLegend	9822	FITC	1:100
CD3	17A2	BioLegend	100241	BV711	1:100
CD3	17A2	BioLegend	100236	APC	1:100
CD3	17A2	BioLegend	100225	BV570	1:100
CD4	GK1.5	BioLegend	100434	PERCP-CY5.5	1:100
CD4	RM4-5	BioLegend	100548	BV605	1:100
CD8a	53-6.7	BioLegend	100752	BV510	1:100
CD8a	53-6.7	BioLegend	100744	BV605	1:100
CD8a	QA17A07	BioLegend	155022	AF700	1:100
CD25	PC61	BioLegend	102008	PE	1:100
CD25	3C7	BioLegend	101904	PE	1:100
CD25	3C7	BioLegend	101916	PE-CY7	1:100
FOXP3	FJK-16S	eBioscience	48-5773-82	EF450	1:50
CD11b	M1/70	BioLegend	101235	BV421	1:100
CD11b	M1/70	BioLegend	101256	PE-Dazzle 594	1:100
CD44	IM7	BioLegend	103040	BV421	1:100
CD44	IM7	BioLegend	103049	BV650	1:100
CD62L	MEL-14	BioLegend	104440	BV785	1:100
CD45RB	C363-16A	BioLegend	103317	PE-CY7	1:100
CD177	Y127	BD Biosciences	566599	AF647	1:100
CD1D	1B1	eBioscience	62-001-182	SB436	1:100
CD80	16-10A1	BD Biosciences	746775	BV480	1:100
TIM3	5D12	BD Biosciences	747625	BV510	1:100
F4/80	BM8	BioLegend	123149	BV650	1:100
LY6C	HK1.4	BioLegend	128037	BV711	1:100
LY6G	1A8	BioLegend	127606	FITC	1:100
MHCII I-A/I-E	2G9	BD Biosciences	565254	BB515	1:200
PD1	29F.1A12	BioLegend	135206	PE	1:100
Ki-67	16A8	BioLegend	652424	PERCP-CY5.5	1:100
B220	RA3-6B2	eBioscience	46-0452-82	PERCP-EF710	1:100
CD11C	N418	BioLegend	117318	PE-CY7	1:100
CD19	6D5	BioLegend	115532	PERCP	1:100
GITR	YGITR765	BioLegend	120222	PE-CY7	1:100
FC (Human)	Clone	Company	Cat#	Color	Dilution
CD45	HI30	BioLegend	982316	FITC	1:100
CD45	HI30	BioLegend	304036	BV510	1:100
CD3	HIT3a	BioLegend	300312	APC	1:100
CD3	HIT3a	BioLegend	300310	PE-CY5	1:100
CD4	RPA-T4	BioLegend	300556	BV605	1:100
CD4	OKT4	BioLegend	317433	BV421	1:100
CD8	SK1	BioLegend	344760	SB550	1:100
CD8	RPA-T8	BioLegend	301044	BV711	1:100
CD25	BC96	BioLegend	302622	AF700	1:100
CD25	M-A251	BioLegend	356108	PE-CY7	1:100
CD127	A019D5	BioLegend	351348	APC-CY7	1:100
CD127	A019D5	BioLegend	351316	APC	1:100
FOXP3	259D	BioLegend	320216	Pacific Blue	1:50
FOXP3	259D/C7	BD Biosciences	560047	AF488	1:50
CD44	IM7	BioLegend	103049	BV650	1:100
CD45RA	HI100	BioLegend	304140	BV785	1:100
CD62L	DREG-56	BioLegend	304822	PE-CY7	1:100
CD177	MEM166	BioLegend	315806	PE	1:100
CD11b	M1/70	BioLegend	101256	PE-Dazzle 594	1:100
Blocking experiment	Clone	Company	Cat#	Color	Concentration
CD177	MEM166	BioLegend	315802	Not Applicable	5 ug/ml
CD177	1171A	R&D SYSTEM	MAB8186	Not Applicable	5 ug/ml
CD177	Polyclonal	LSBIO	LS-C295622-100	Not Applicable	5 ug/ml
Mouse IgG	G3A1	Cell Signaling	5415S	Not Applicable	5 ug/ml
CD3e	145-2C11	BioLegend	553058	Not Applicable	1 ug/ml
CD28	37.51	eBioscience	16-0281-82	Not Applicable	1.5 ug/ml

Supplementary Table 1. List of antibodies in this paper.

Real-time PCR primers	
Gene	Primer sequence
mCd177-1	CCGGGAGAATATGGAGACAC
mCd177-2	CGCTGCTGCTCATAGACGTA
mPpia-1	CAGTGCTCAGAGCTCGAAAGT
mPpia-2	GTGTTCTTCGACATCACGGC
mFoxp3-1	CTCGTCTGAAGGCAGAGTCA
mFoxp3-2	TGGCAGAGAGGTATTGAGGG
**Genotyping Primers**	
Gene	Primer sequence
mCd177-1 5’ loxp	GTGTTGCGTTTCCTGCTTG
mCd177-2 5’ loxp	CTGGTTACCTTATGCCACTCC
mCd177-1 3’ loxp	GGGTTGCCAAGACTTGATAATG
mCd177-2 3’ loxp	AGGTGAGACACTAGAGAAGAGG

Supplementary Table 2. List of primers used for real-time PCR.
