# Supplementary material for: CD177 modulates the function and homeostasis of tumor-infiltrating regulatory T cells
Source: Nat Commun. 2021 Oct 1;12:5764. doi: 10.1038/s41467-021-26091-4 (PMC8486774; doi:10.1038/s41467-021-26091-4)
Supplement: Supplementary file 3 — Reporting Summary [file 41467_2021_26091_MOESM3_ESM.pdf]

natureresearch

Corresponding author(s): Weizhou Zhang

Last updated by author(s): Mar 2, 2021

Reporting Summary

Nature Research wishes to improve the reproducibility of the work that we publish. This form provides structure for consistency and transparency in reporting. For further information on Nature Research policies, see Authors & Referees and the Editorial Policy Checklist .

Statistics

For all statistical analyses, confirm that the following items are present in the figure legend, table legend, main text, or Methods section.

n/a	Confirmed	
☐	☒	The exact sample size (*n*) for each experimental group/condition, given as a discrete number and unit of measurement
☐	☒	A statement on whether measurements were taken from distinct samples or whether the same sample was measured repeatedly
☐	☒	The statistical test(s) used AND whether they are one- or two-sided<br>*Only common tests should be described solely by name; describe more complex techniques in the Methods section.*
☐	☒	A description of all covariates tested
☐	☒	A description of any assumptions or corrections, such as tests of normality and adjustment for multiple comparisons
☐	☒	A full description of the statistical parameters including central tendency (e.g. means) or other basic estimates (e.g. regression coefficient) AND variation (e.g. standard deviation) or associated estimates of uncertainty (e.g. confidence intervals)
☐	☒	For null hypothesis testing, the test statistic (e.g. F, t, r) with confidence intervals, effect sizes, degrees of freedom and P value noted<br>*Give P values as exact values whenever suitable.*
☒	☐	For Bayesian analysis, information on the choice of priors and Markov chain Monte Carlo settings
☒	☐	For hierarchical and complex designs, identification of the appropriate level for tests and full reporting of outcomes
☒	☐	Estimates of effect sizes (e.g. Cohen's *d*, Pearson's *r*), indicating how they were calculated

Our web collection on statistics for biologists contains articles on many of the points above.

Software and code

Policy information about availability of computer code

Data collection	All codes for single cell analysis were deposited into GibHub by Dr. Borcherding. Link - https://github.com/ncborcherding
Data analysis	Data analysis was done by the Dr. Borcherding who developed all the codes and statistical analysis. Software used: FlowJo_v10.6.1; GraphPad Prism 8.3.0; R (v3.5.1); Seurat R package (v2.3.4); sleuth R package (v0.30.0); monocle 2 R Package; SingleR R package V0.2.0; CellRanger v2.2 pipeline were used for all analysis. All analyses were further validated by Dr. Bacher in the author list who is an expert in bioinformatics and statistics.

For manuscripts utilizing custom algorithms or software that are central to the research but not yet described in published literature, software must be made available to editors/reviewers. We strongly encourage code deposition in a community repository (e.g. GitHub). See the Nature Research guidelines for submitting code & software for further information.

Data

Policy information about availability of data

All manuscripts must include a data availability statement. This statement should provide the following information, where applicable:

- Accession codes, unique identifiers, or web links for publicly available datasets
- A list of figures that have associated raw data
- A description of any restrictions on data availability

All genome data are available via GSE datasets with links provided. We included new analysis from publicly available datasets including: GSE89225, GSE98638, PRJEB11844. The single-cell RNA sequencing result for renal cancer is deposited as GSE121638 and the TI-Tregs from MC38 tumor model is deposited as GSE150420, all with links in the data availability section. TCGA datasets were downloaded for the TCGA website or UCSC Xena Browser (https://xena.ucsc.edu/) including KIRC/ccRCC, breast cancer, melanoma and 21 other cancer RNA sequencing datasets and clinical parameters. All modified codes related to SCRC analysis are deposited at https://github.com/ncborcherding. All newly developed mouse models will be make available upon request. All source data associated with statistics within all relevant Figures are provided and the original data will be made available upon request.

Field-specific reporting

Please select the one below that is the best fit for your research. If you are not sure, read the appropriate sections before making your selection.

- [x] Life sciences
- [ ] Behavioural & social sciences
- [ ] Ecological, evolutionary & environmental sciences

For a reference copy of the document with all sections, see nature.com/documents/nr-reporting-summary-flat.pdf

Life sciences study design

All studies must disclose on these points even when the disclosure is negative.

Sample size	Sample size is used depending on individual experiments, based on variation, statistical power and the nature of the experiments. For example tumor studies with expectation of 1 fold variation and power 0.8, we estimate 6-7 mice per group. For other experiments, we don't have estimate of group variations and expected fold change. We recorded data as we performed individual experiments, and used at least 3 biological replicates to calculate P values of various statistical methods, using GraphPad Prism 8.3.0. For tumor studies, a two-way ANOVA was used; for two group comparisons, unpaired T test was used; for single cell RNAseq data or RNAseq data, we used Bonferroni adjusted P values. We normally use P < 0.05 to be considered statistically significant.
Data exclusions	All data is included, unless flow data when no cells were detected due to technical error.
Replication	All experiments are labeled for biological replicates.
Randomization	All animal experiments were randomized. For mice receiving different treatments, we routinely put all mice into the same container and separate them out randomly into groups for different treatment. For other experiments related to flow cytometry, the randomization was at the stage of mice separation as above. For human samples, randomization didn't apply and we used whatever samples we could get from collection and compare between groups. For Treg suppression assay, tumors are randomly combined for each group to achieve sufficient number of Treg cells for suppression; to quantitate cell numbers between tumor groups, we used all tumors that were able to be analyzed during the experiments to avoid selection bias. When the allocation was not random in experimental groups, the covariate consideration is not relevant since all experimental groups are determined by the initial assignment from the tumor-bearing mice (randomly assigned) and not excluded.
Blinding	All tumor studies are double blinded. The treatment or grouping is from the leading postdoc/technician and the tumor measurement is from an assisting different postdoc/technician. To avoid technical error, one person sticks to one particularly data acquisition. For example, the same person will continue to monitor tumor growth for the same cohort of experiments. For other experiments, blinding is impossible and there is no step that can introduce personal bias.

Reporting for specific materials, systems and methods

We require information from authors about some types of materials, experimental systems and methods used in many studies. Here, indicate whether each material, system or method listed is relevant to your study. If you are not sure if a list item applies to your research, read the appropriate section before selecting a response.

Materials & experimental systems

n/a	Involved in the study	
☐	☒	Antibodies
☐	☒	Eukaryotic cell lines
☒	☐	Palaeontology
☐	☒	Animals and other organisms
☐	☒	Human research participants
☒	☐	Clinical data

Methods

n/a	Involved in the study	
☒	☐	ChIP-seq
☐	☒	Flow cytometry
☒	☐	MRI-based neuroimaging

Antibodies

Antibodies used

We used many antibodies, all from well validated companies, such as flow antibodies are mostly from Biolegend (BL), ebioscience (eB), BD Bioscience (BD), unless otherwise noted: FC (mouse) Clone Catalogue number
CD45.1 A20 BL:110730
CD45.2 30-F11 eB: 58-0451-82
CD45.2 104 BL:109822
CD3 17A2 BL:100241, 100236, 100225
CD4 GK1.5 BL:100434
CD4 RM4-5 BL:100548
CD8a 53-6.7 BL:100752, 100744, 155022
CD25 PC61 BL:102008

CD25 3C7 BL:101904, 101916
FOXP3 FJK-16S eB: 48-5773-82
CD11b M1/70 BL:101235, 101256
CD44 IM7 BL:103040, 103049
CD62L MEL-14 BL:104440
CD45RB C363-16A BL:103317
CD177 Y127 BD:566599
CD1D 1B1 eB: 62-001-182
CD80 B7-1 BL:746775
TIM3 5D12 BL:747625
F4/80 BM8 BL:123149
LY6C HK1.4 BL:128037
LY6G 1A8 BL:127606
MHCII I-A/I-E 2G9 BD:565254
PD1 29F.1A12 BL:135206
Ki-67 16A8 BL:652424
B220 RA3-6B2 eB: 46-0452-82
CD11C N418 BL: 117318
CD19 6D5 BL:115532
GITR YGITR765 BL:120222

FC (Human) Clone Catalogue number
CD45 HI30 BL:982316, 304036
CD3 HIT3a BL:300312, 300310
CD4 RPA-T4 BL:300556
CD4 OKT4 BL:317433
CD8 SK1 BL:344760
CD8 RPA-T8 BL:301044
CD25 BC96 BL:302622
CD25 M-A251 BL:356108
CD127 A019D5 BL:351348, 351316
FOXP3 259D BL:320216
FOXP3 259D/C7 BL:560047
CD44 IM7 BL:103049
CD45RA HI100 BL:304140
CD62L DREG-56 BL:304822
CD177 MEM166 BL:315806
CD11b M1/70 BL:101256

Blocking experiment Clone Catalogue number
CD177 MEM166 BL:315802
CD177 1171A R&D: MAB8186
CD177 Polyclonal LS-C295622-100
Mouse IgG G3A1 CS: 5415S
CD3e 145-2C11 BL: 553058
CD28 37.51 eB: 16-0281-82
All standard staining procedure of 1:100 or 1:200 dilution.

Validation

Most of these antibodies are used hundreds of times in literature and validated from a lot publications. Company validates each batch before selling. The lab routinely validates new antibodies using various approaches.

FC (mouse) clone Application notes Validation Reference

FC (mouse) Clone Catalogue number Application notes Validation Reference

CD45.1 A20 BL:110730 The A20 antibody does not react with leukocytes or mouse cells expressing the CD45.2 alloantigen. Flow Cytometry - Qaulity tested Phan TG, et al. 2007. Nature Immunol. 8:992

CD45.2 30-F11 eB: 58-0451-82 The 30-F11 monoclonal antibody reacts with all isoforms of mouse CD45 Flow Cytometry - Qaulity tested Huang, Jian, et al. 2019.Nature communications 10.1: 1-13

CD45.2 104 BL:109822 The 104 antibody does not react with mouse cells expressing the CD45.1 alloantigen Flow Cytometry - Qaulity tested Kohlmeier JE, et al. 2008. Immunity. 29:101

CD3 17A2 BL:100241, 100236, 100225 Flow Cytometry - Qaulity tested Haikala HM, et al. 2019. Nat Commun. 10:620

CD4 GK1.5 BL:100434 Additional reported applications (for the relevant formats) include: blocking of CD4+ T cell activation, thymocyte costimulation, in vitro and in vivo depletion, blocking of egg-sperm cell adhesion, immunohistochemical staining of acetone-fixed frozen sections, and immunoprecipitation Flow Cytometry - Qaulity tested Bing Wu et al. 2018. Immunity. 49 (5):886-898

CD4 RM4-5 BL:100548 The RM4-5 antibody blocks the binding of GK1.5 antibody and H129.19 antibody to CD4+ T cells, but not RM4-4 antibody Flow Cytometry - Qaulity tested Matsumoto M, et al. 2007.J. Immunol.178:2499

CD8a 53-6.7 BL:100752, 100744, 155022 Clone 53-6.7 antibody competes with clone 5H10-1 antibody for binding to thymocytes

Flow Cytometry - Qaulity tested Ko SY, et al. 2005. J. Immunol. 175:3309

CD25 PC61 BL:102008 Additional reported applications (for the relevant formats) include: immunoprecipitation, in vitro blocking of IL-2 binding to low- and high-affinity receptors, growth inhibition of IL-2-dependent T-cell lines, in vivo depletion of CD25+CD4 + Treg cells, and immunohistochemical staining of acetone-fixed frozen sections Flow Cytometry - Qaulity tested León-Ponte M, et al. 2007. Blood 109:3139

CD25 3C7 BL:101904, 101916 Additional reported applications (for the relevant formats) include: in vitro blocking of IL-2 binding to low- and high-affinity receptors and immunohistochemical staining of acetone-fixed frozen sections. Flow Cytometry - Qaulity tested Uchimura T et al. 2018. Immunity. 49(6):1049-1061

FOXP3 FJK-16S eB: 48-5773-82 This FJK-16s antibody has been reported for use in intracellular staining followed by flow cytometric analysis Flow Cytometry - Qaulity tested Baumann, Daniel, et al. 2020. Nature communications 11.1 (2020): 1-18

CD11b M1/70 BL:101235, 101256 Additional reported applications (for relevant formats of this clone) include: immunoprecipitation, in vitro blocking, depletion, immunofluorescence microscopy, and immunohistochemistry of acetone-fixed frozen sections Flow Cytometry - Qaulity tested Desai P, et al. 2021. Cell. 184(5):1214-1231.e16

CD44 IM7 BL:103040, 103049 Clone IM7 has been reported to recognize an epitope common to alloantigens and all isoforms of CD44 that is located between amino acids 145 and 186. Flow Cytometry - Qaulity tested Demircioglu F, et al. 2020. Nat Commun. 11:1290

CD62L MEL-14 BL:104440 Additional reported applications (for the relevant formats) include: immunoprecipitation, complement-dependent cytotoxicity, in vivo and in vitro blocking of adhesion, and immunohistochemical staining of acetone-fixed frozen sections and zinc-fixed paraffin-embedded sections Flow Cytometry - Qaulity tested Preglej T, et al. 2020. JCI Insight. 5(4)

CD45RB C363-16A BL:103317 Additional reported applications (for the relevant formats) include: immunoprecipitation, immunohistochemistry of acetone-fixed frozen sections and formalin-fixed paraffin-embedded sections. Flow Cytometry - Qaulity tested Norian LA and Allen PM. 2004. J. Immunol. 173:835

CD177 Y127 BD:566599 The Y127 monoclonal antibody specifically recognizes CD177 which is also known as Pdp3 Flow Cytometry - Qaulity tested Xie Q, Protein Cell. 2015; 6(2):117-26.

CD1D 1B1 eB: 62-001-182 Flow Cytometry - Qaulity tested

CD80 B7-1 BL:746775 The 16-10A1 monoclonal antibody specifically recognizes CD80 (B7-1) Flow Cytometry - Qaulity tested Sojka DK, J Immunol. 2000; 164(12):6230-6236

TIM3 5D12 BL:747625 The 5D12 monoclonal antibody specifically recognizes CD366 which is also known as TIM-3 (T-cell immunoglobulin and mucin domain-containing 3) or T-cell membrane protein 3 Flow Cytometry - Qaulity tested Veenstra RG, Blood. 2012; 120(3):682-90

F4/80 BM8 BL:123149 Additional reported applications (for the relevant formats) include: immunohistochemical staining of acetone-fixed frozen sections and formalin-fixed paraffin-embedded sections and Western blotting. Flow Cytometry - Qaulity tested Campesato LF, et al. 2020. Nat Commun. 3.24375

LY6C HK1.4 BL:128037 Clone HK1.4 does not block the binding of clone RB6-8C58 Flow Cytometry - Qaulity tested Komuczki J, et al. 2019. Immunity. 50:1289

LY6G 1A8 BL:127606 While 1A8 recognizes only Ly-6G, clone RB6-8C5 recognizes both Ly-6G and Ly-6C Flow Cytometry - Qaulity tested Li Z et al. 2018. Immunity. 49(4):640-653

MHCII I-A/I-E 2G9 BD:565254 The 2G9 monoclonal antibody reacts with the I-Ad and I-Ed MHC class II alloantigens Flow Cytometry - Qaulity tested Farquhar CA, Eur J Immunol. 2010; 40(6):1728-1737

PD1 29F.1A12 BL:135206 Additional reported applications (for the relevant formats) include: immunohistochemical staining of acetone-fixed frozen tissue and in vivo blocking of PD-1 binding to its ligands Flow Cytometry - Qaulity tested Kim CJ, et al. 2018. Immunity. 49:1034

Ki-67 16A8 BL:652424 Flow Cytometry - Qaulity tested Kumagai S, et al. 2020. Immunity. 53(1):187-203.e8

B220 RA3-6B2 eB: 46-0452-82 "The RA3-6B2 monoclonal antibody reacts with exon A-restricted isoform of mouse CD45, a 220 kDa surfacemolecule" Flow Cytometry - Qaulity tested Lam, Jeffrey, et al. Nature communications 9.1 (2018): 1-14

CD11C N418 BL: 117318 Additional reported applications (for the relevant formats) include: immunoprecipitation, immunohistochemical staining of acetone-fixed frozen sections, and immunofluorescence microscopy Flow Cytometry - Qaulity tested Yuzhu Hou et al. 2018. Immunity. 49(3):490-503

CD19 6D5 BL:115532 Additional reported applications (for the relevant formats) include: immunofluorescence Flow Cytometry - Qaulity tested Miyai T, et al. 2014. Proc Natl Acad Sci U S A. 111:1780.

GITR YGITR765 BL:120222 The LEAF™ purified antibody (Endotoxin <0.1 EU/μg, Azide-Free, 0.2 μm filtered) is recommended for functional assays Flow Cytometry - Qaulity tested Kumagai S, et al. 2020. Immunity. 53(1):187-203.e8

FC (Human) Clone Catalogue number Application notes Validation Reference

CD45 HI30 BL:982316, 304036 Additional reported applications (for the relevant formats) include: immunohistochemical staining of acetone-fixed frozen tissue sections and formalin-fixed paraffin-embedded tissue sections, inhibition of CD45 functions, immunofluorescence, and Western blotting Flow Cytometry - Qaulity tested Affo S, et al. 2021. Cancer Cell. 39 (6):866-882.e11

CD3 HIT3a BL:300312, 300310 Additional reported (for the relevant formats) applications include: immunohistochemical staining of acetone-fixed frozen sections, immunoprecipitation, and activation of T lymphocytes. Flow Cytometry - Qaulity tested Ho T, et al. 2016. Blood. 128: 1671 - 1678

CD4 RPA-T4 BL:300556 The RPA-T4 antibody binds to the D1 domain of CD4 (CDR1 and CDR3 epitopes) and can block HIV gp120 binding and inhibit syncytia formation Flow Cytometry - Qaulity tested Juno JA, et al. 2020. Nat Med. 26:1428

CD4 OKT4 BL:317433 The OKT4 antibody binds to the D3 domain of CD4 and does not block HIV binding Flow Cytometry - Qaulity tested Iwata A, et al. 2017. Nat Immunol. 18:56

CD8 SK1 BL:344760 Clone SK1 recognizes the a chain of CD8 Flow Cytometry - Qaulity tested Peterson VM, et al. 2017. Nat. Biotechnol. 35:936

CD8 RPA-T8 BL:301044 The RPA-T8 antibody does not block the binding of HIT8a antibody to CD8a Flow Cytometry - Qaulity tested Gurusamy D, et al. 2020. Cancer Cell. 37(6):818-833.e9

CD25 BC96 BL:302622 Additional reported applications include: immunocytochemistry Flow Cytometry - Qaulity tested Wang J, et al. 2009. J Immunol. 183:4119
CD25 M-A251 BL:356108 Additional reported applications (for the relevant formats) include: immunohistochemical staining of paraformaldehyde fixed frozen sections Flow Cytometry - Qaulity tested Kreutmair S, et al. 2021. Immunity
CD127 A019D5 BL:351348, 351316 Additional reported (for the relevant formats) application: proteogenomics Flow Cytometry - Qaulity tested Caduff N, et al. 2021. Cell Reports. 35(5):109056
FOXP3 259D BL:320216 Additional reported applications (for the relevant formats) include: Western blotting, and immunohistochemical staining of acetone-fixed frozen sections and formalin-fixed paraffin-embedded sections Flow Cytometry - Qaulity tested Ickrath P, et al. 2018. Int J Mol Med. 42:1116
FOXP3 259D/C7 BL:560047 The 259D/C7 monoclonal antibody specifically recognizes human Forkhead box protein P3 (FoxP3) that is also known as Scurfin Flow Cytometry - Qaulity tested Brunkow ME, Nat Genet. 2001; 27(1):68-73.
CD44 IM7 BL:103049 Clone IM7 has been reported to recognize an epitope common to alloantigens and all isoforms of CD44 Flow Cytometry - Qaulity tested Dikiy S, et al. 2021. Immunity. 54(5):931-946.e11
CD45RA HI100 BL:304140 Additional reported applications (for relevant formats of this clone) include: inhibition of CD45 functions, immunohistochemical staining of frozen tissue sections and formalin-fixed paraffin-embedded tissue sections, and immunocytochemistry Flow Cytometry - Qaulity tested Kariminia A, et al. 2016. Blood. 127: 3082 - 3091
CD62L DREG-56 BL:304822 Additional reported applications (for the relevant formats) include: Western blotting and in vitro blocking of lymphocytes binding to high endothelial venules (HEV) Flow Cytometry - Qaulity tested Giamarellos-Bourboulis EJ, et al. 2020. Cell. 183(2):315-323.e
CD177 MEM166 BL:315806 Additional reported applications (for the relevant formats) include: immunoprecipitation, Western blotting, and immunofluorescence Flow Cytometry - Qaulity tested Korkmaz B, et al. 2008. J. Biol. Chem. 283:35976.
CD11b M1/70 BL:101256 Additional reported applications (for relevant formats of this clone) include: immunoprecipitation, in vitro blocking, depletion, immunofluorescence microscopy, and immunohistochemistry of acetone-fixed frozen sections Flow Cytometry - Qaulity tested Desai P, et al. 2021. Cell. 184(5):1214-1231.e16.

Blocking experiment Clone Catalogue number Application notes Validation Reference
CD177 MEM166 BL:315802 Additional reported applications (for the relevant formats) include: immunoprecipitation, Western blotting, and immunofluorescence, IHC Flow Cytometry - Qaulity tested von Vietinghoff S, et al. 2007. Blood 109:4487
CD177 1171A R&D: MAB8186 Flow Cytometry - Qaulity tested van Timmeren, M.M. and P. Heeringa (2012) Curr. Opin. Rheumatol. 24:8
CD177 Polyclonal LS-C295622-100 The antibody is a rabbit polyclonal antibody raised against NB1. Validated for Western blot and IHC
Mouse IgG G3A1 CS: 5415S Flow Cytometry - Qaulity tested Wright, Griffin, International journal of molecular sciences 22.11 (2021): 5475.
CD3e 145-2C11 BL: 553058 The 145-2C11 monoclonal antibody specifically binds to the 25-kDa ε chain of the T-cell receptor-associated CD3 complex that is expressed on thymocytes, mature T lymphocytes, and NK-T cells. The cytoplasmic domain of CD3e participates in the signal transduction events that activate several cellular biochemical pathways as a result of antigen recognition. Flow Cytometry - Qaulity tested Chai JG, Lechler RI. Int Immunol. 1997; 9(7):935-944.
CD28 37.51 eB: 16-0281-82 The 37.51 monoclonal antibody reacts with the mouse CD28 molecule, a 45 kDa homodimer expressed by thymocytes, mature T cells and NK cells. CD28 is a ligand for CD80 (B7-1) and CD86 (B7-2) and is a potent costimulator of T cells Flow Cytometry - Qaulity tested Wright, Griffin, International journal of molecular sciences 22.11 (2021): 5475.

Eukaryotic cell lines

Policy information about cell lines

Cell line source(s)

MC38 is from Dr. Zhu,Yuwen laboratory in the author list. PY8119 is from Dr. Lesley before she provided to ATCC.

Authentication

All cells are frozen a large batch from ATCC or from collaborators. They haven't been passaged a lot. We normally use morphological feature for distinct cell lines, ability to form tumors and histology of these tumors. We only use cell lines within 6 passages from ATCC if possible. They are not authenticated using STR profiling in the laboratory.

Mycoplasma contamination

All cells were screened by mouse pathogen panels and all are free of mycoplasma or other mouse pathogens. Results can be provided.

Commonly misidentified lines
(See ICLAC register)

No commonly misidentified cell lines were used in the study.

Animals and other organisms

Policy information about studies involving animals; ARRIVE guidelines recommended for reporting animal research

Laboratory animals

C57BL/6 Mice of C57BL/6 background, including wild type males and females, CD177 germ-line knock out mice in the C57BL/6 background, CD177fl/fl mice of C57BL/6, CD177fl/fl/Foxp3Cre/YFP mice. We used 7-8 week old mice for tumor study and euthanized the mice between 12-16 weeks of age. All animal studies were approved by the University of Florida Institutional Animal Care and Use Committee (IACUC) under protocol 201810399. All animals were housed in a pathogen-free Association for Assessment and Accreditation of Laboratory Animal Care accredited facility at the University of Florida and performed in accordance with IACUC guidelines. The room temperature is 21.1–23.3 °C with an acceptable daily fluctuation of 2 °C. Typically the room is 22.2 °C all the time. The humidity set point is 50% but can vary ±15% daily depending on the weather. The

	photoperiod is 12:12 and the light intensity range is 15.6–25.8 FC.
Wild animals	No wild animals were used in the study.
Field-collected samples	No field collected samples were used in the study.
Ethics oversight	All animal studies were approved by the University of Florida Institutional Animal Care and Use Committee (IACUC) with an approved protocol number 201810399.

Note that full information on the approval of the study protocol must also be provided in the manuscript.

Human research participants

Policy information about studies involving human research participants

Population characteristics	The patients were males with an age range of 67 to 74 years old. Tumor grades were histologically determined by a pathologist. Primary tumor stages for Patient 1 and Patient 2 were reported as pT1b without extension, while Patient 3 was reported as pT3a with renal vein invasion. Paired blood and primary ccRCC along with matched normal kidney parenchyma samples were obtained from the University of Iowa Tissue Procurement Core and GUMER repository through the Holden Comprehensive Cancer Center from de-identified three subjects previously provided written consent approved by the University of Iowa Institutional Review Board (IRB) under the IRB number 201304826 and conducted under the Declaration of Helsinki Principles.
Recruitment	Patients recruitment was through the University of Iowa Tissue Procurement Core and GUMER repository from the Holden Comprehensive Cancer Center, de-identified with written consents. There is no selection and we only chose three renal cell carcinoma patients with 500 mg of tissues to get enough tumor infiltrating immune cells for the study.
Ethics oversight	The current study was approved by the University of Iowa Institutional Review Board (IRB) under the IRB number 201304826 and conducted under the Declaration of Helsinki Principles. De-identified renal cancer patients were recruited by Dr. Yousef Zakharia in the Department of Internal Medicine at the University of Iowa. Some human tissues were also collected from patients undergoing surgical resection after informed consent and were supplied as de-identified samples to Dr. Zhu laboratory with IRB approval (IRB number: 13-0315) or to Dr. Zhang with IRB approval (IRB number: IRB201901677). Additional human specimens were collected from the Renji Hospital, Shanghai Jiao Tong University School of Medicine in an anonymous manner to the Dr. Xuefeng Wu laboratory for cell preparation and T cell suppression assays, approved by the University Human Subject Protection Committee. All human protocols were carefully read and evaluated by the full review panels from the corresponding University Institutional Review Board (IRB), following annual review for the progress, ethics and remaining tissue procurement.

Note that full information on the approval of the study protocol must also be provided in the manuscript.

Flow Cytometry

Plots

Confirm that:

- [x] The axis labels state the marker and fluorochrome used (e.g. CD4-FITC).
- [x] The axis scales are clearly visible. Include numbers along axes only for bottom left plot of group (a 'group' is an analysis of identical markers).
- [x] All plots are contour plots with outliers or pseudocolor plots.
- [x] A numerical value for number of cells or percentage (with statistics) is provided.

Methodology

Sample preparation	Tumors, spleen, lymph nodes, bloods and thymus from mice were routinely used. Tumors were excised and approximately 200 mg of tumor tissue were enzymatically and mechanically digested using the mouse Tumor Dissociation Kit (Miltenyi Biotec) to obtain a single cell suspension. Human tumor samples and sections were enzymatically and mechanically digested using the human Tumor Dissociation Kit (Miltenyi Biotec) to obtain single cell suspension. Red blood cells were lysed using ACK lysis buffer and mononuclear cells were isolated by density gradient using SepMate Tubes (StemCell Technologies) and Lymphoprep density gradient media (StemCell Technologies). Cells were then washed and incubated with combinations of antibodies for staining and intracellular staining.
Instrument	BD LSRII, Aria II, Aurora Cytek
Software	Flowjo
Cell population abundance	Cell populations, percentages and numbers were determined by individual experiments. For Tregs in human tissues, with our stringent gating on live cells, lymphocytes, and other parameters, human cancers may only have very limited number of Tregs.
Gating strategy	Examples were included for SSC/FSC gating. Negative/positive-gating was based on non-staining controls for well established antibodies and FMO for new antibodies used.

- [x] Tick this box to confirm that a figure exemplifying the gating strategy is provided in the Supplementary Information.
