# Supplementary material for: CD177 modulates the function and homeostasis of tumor-infiltrating regulatory T cells
Source: Nat Commun. 2021 Oct 1;12:5764. doi: 10.1038/s41467-021-26091-4 (PMC8486774; doi:10.1038/s41467-021-26091-4)
Supplement: Supplementary file 4 — Description of Additional Supplementary Files [file 41467_2021_26091_MOESM4_ESM.pdf]

Description of Additional Supplementary Files

Supplementary Data 1: DEGs between TI-Treg cells and PB-Treg cells from ccRCC single cell RNAseq.

Wilcoxon rank sum test with p-values adjusted using the Bonferroni method.

Supplementary Data 2: DEGs between TI-Treg cells and PB-Treg cells from HCC single cell RNAseq

Wilcoxon rank sum test with p-values adjusted using the Bonferroni method.

Supplementary Data 3: All genes for pseudotime analysis in Figure 2B.

Supplementary Data 4: Treg gene signatures from ccRCC single cell RNAseq used for filtering in Figure 3.
